# Transcriptome profiling of *Brassica napus* stem sections in relation to differences in lignin content

**DOI:** 10.1186/s12864-018-4645-6

**Published:** 2018-04-16

**Authors:** Zakir Hossain, Bhinu V.-S. Pillai, Margaret Y. Gruber, Min Yu, Lisa Amyot, Abdelali Hannoufa

**Affiliations:** 1 Agriculture and Agri-Food Canada, London Research and Development Centre, 1391 Sandford Street, London, ON N5V 4T3 Canada; 2 Agriculture and Agri-Food Canada, Swift Current Research and Development Centre, 1 Airport Road, Swift Current, SK S9H 3X2 Canada; 3 Agriculture and Agri-Food Canada, Agassiz Research and Development Centre, 6947 Highway 7, Post Office Box 1000, Agassiz, BC V0M 1A0 Canada; 4 Agriculture and Agri-Food Canada, Saskatoon Research and Development Centre, 107 Science Place, Saskatoon, SK S7N 0X2 Canada

**Keywords:** Biomass, *Brassica*, Cell wall, Stem lignin, Microarray, Transcription factors

## Abstract

**Background:**

Brassica crops are cultivated widely for human consumption and animal feed purposes, and oilseed rape/canola (*Brassica napus* and *rapa*) is the second most important oilseed worldwide. Because of its natural diversity and genetic complexity, genomics studies on oilseed rape will be a useful resource base to modify the quantity and quality of biomass in various crops, and therefore, should have a positive impact on lignocellulosic biofuel production. The objective of this study was to perform microarray analysis on two variable lignin containing oilseed rape cultivars to target novel genes and transcription factors of importance in Brassica lignin regulation for applied research.

**Results:**

To gain insight into the molecular networks controlling cell wall biosynthetic and regulatory events, we conducted lignin and microarray analysis of top and basal stem sections of brown seeded *Brassica napus* DH12075 and yellow seeded YN01–429 cultivars. A total of 9500 genes were differentially expressed 2-fold or higher in the stem between the cultivars, with a higher number of expressed genes in the basal section. Of the upregulated genes, many were transcription factors and a considerable number of these were associated with secondary wall synthesis and lignification in *B. napus* and other plant species. The three largest groups of transcription factors with differential expression were C2H2 and C3HC4 zinc fingers and bHLH. A significant number of genes related to lignin and carbohydrate metabolism also showed differential expression patterns between the stem sections of the two cultivars. Within the same cultivar, the number of upregulated genes was higher in the top section relative to the basal one.

**Conclusion:**

In this study, we identified and established expression patterns of many new genes likely involved in cell wall biosynthesis and regulation. Some genes with known roles in other biochemical pathways were also identified to have a potential role in cell wall biosynthesis. This stem transcriptome profiling will allow for selecting novel regulatory and structural genes for functional characterization, a strategy which may provide tools for modifying cell wall composition to facilitate fermentation for biofuel production.

**Electronic supplementary material:**

The online version of this article (10.1186/s12864-018-4645-6) contains supplementary material, which is available to authorized users.

## Background

Limiting sources of fossil energy resources and potential climate change underline increasing worldwide economic interest and scientific focus on renewable biofuel. Lignocellulosic biomass is abundant in nature and can be used as sustainable and renewable feedstocks for biofuel production. Grasses, such as maize, sorghum, switchgrass and Miscanthus, and fast-growing trees, such as poplar and willow are potential sources of lignocellulosic biomass. Crop residues collected from the production of major food crops such as canola, maize, rice and wheat can also be used as raw material for biofuel. Brassica crops are among the oldest cultivated plants, and oilseed rape/canola (*Brassica napus* and *rapa*) is currently the second most important oilseed worldwide [1]. Because of its wide spread cultivation, natural diversity and genetic complexity [2, 3], genomic studies on oilseed rape are a useful resource base from which to modify the quantity and quality of biomass in various crops, and hence, should have a positive impact on lignocellulosic biofuel production.

Crop residues are a rich source of plant biomass, which is mainly composed of plant cell walls. The major components of plant cell walls are cellulose, hemicellulose, pectin and lignin. Cellulose is the world’s most abundant biopolymer [4] and can be used as a sustainable and renewable feedstock for biofuels [5, 6]. However, this process is inhibited by the presence of lignin [7], a major component of secondary cell walls, which provides structural support to plants but also acts as an obstacle to glucose fermentation by increasing the recalcitrance of cell wall digestibility [8]. Moreover, lignin can adsorb hydrolytic enzymes that are used to generate monosaccharides from lignocellulose, and some lignin degradation products inhibit subsequent fermentation steps [9]. Novel strategies to reduce lignin and/or modify its composition in the plant cell wall are therefore needed, and a clear understanding of cell wall biogenesis-related gene expression with emphasis on lignin biosynthesis and factors regulating its accumulation and polymerization is essential to addressing cell wall recalcitrance.

Although many of the structural genes in lignin biosynthesis have been characterized, additional knowledge on transcription factors controlling this pathway is still needed. Studies have shown developmental regulation of the phenylpropanoid pathway genes by various classes of *trans*-acting factors [10]. Although, most of the genes encoding lignin biosynthetic enzymes have been targets for modification of lignin biosynthesis, only a few studies have taken advantage of the possibility of modulating the *trans*-acting factors involved in phenylpropanoid metabolism. Some promising findings on the role of transcription factors with phenylpropanoid end products, including lignin biosynthesis, have been reported through mutant analysis [11–15], but additional work is still needed in this area. Presumably, altering the expression level of appropriate transcription factors would coordinately affect a group of genes in the pathway. By doing so, it might be possible to avoid deleterious phenotypes associated with the accumulation of pathway intermediates due to single gene/enzyme manipulations [16].

Microarrays have been an important technology for the global analysis of gene expression in plants, including in cell walls, although more recently RNA sequencing has risen in importance. Hertzberg et al. [17] used poplar cDNA arrays to profile changes in gene expression at various stages of secondary xylem differentiation. Ko et al. [18] used short oligonucleotide arrays to identify genes that display preferred expression in secondary xylem and during the transition from primary to secondary growth in *Arabidopsis* stems. Ehlting et al. [19] studied the metabolic, developmental and regulatory events at different stages of vascular and interfascicular fiber differentiation in inflorescence stems of *Arabidopsis* using oligo arrays.

The yellow seeded *B. napus* YN01–429 cultivar was recently developed through classical breeding approaches. It was found to contain significantly reduced seed lignin content relative to the more conventional brown seeded cultivar DH12075 [20, 21]. The yellow-colored trait of Brassica seeds is valuable to Brassica breeders since it is associated with a thinner seed coat and reduced dietary fiber content [21]. Light seed color and low fiber content are believed to share precursors and biochemical pathways leading to lignin and pigment synthesis [22, 23]. Since plant stems represent the major contribution to Brassica plant residue, we analyzed the total lignin content of stem sections of these two unique cultivars. On the basis of differential lignin content, we performed microarray experiments to determine variations in the transcription profiles of different sections of the stem. Analysis of transcript profiles allowed us to categorize genes into specific sets involved in various regulatory, developmental and metabolic processes including transcriptional regulation, carbohydrate metabolism, and lignin and cellulose biosynthesis. By correlating changes in gene expression with changes in the levels of lignin, we hypothesized that it should be possible to derive insights into the regulatory mechanisms of cell wall biosynthesis and to find target transcription factors of importance in Brassica lignin regulation for practical purposes.

## Methods

### Plant materials and growth conditions

Brown seeded *B. napus* DH12075 (designated DH), yellow seeded *B. napus* YN01–429 (designated YN), alfalfa (*Medicago sativa* L.) and *Arabidopsis thaliana* ecotypes and mutant lines were grown in soil in a controlled greenhouse environment (16 h light/8 h dark, 20 °C/17 °C). For *B. napus* stem samples, plants with well-developed siliques were harvested and 40 cm of the stem was selected (excluding the lower most 10 cm). Collected stem samples were divided into 4 segments, 10 cm each, and apical (section 1) and basal sections (section 4) were used for lignin, microarray and qPCR analyses. For Arabidopsis lignin analysis, Arabidopsis plants (42-day-old) were cut at the base of the inflorescence stem and leaves and thin branches were removed, keeping the stem and main/robust branches for analysis.

### Lignin analysis

Lignin content of stem material was determined by thioglycolic acid (TGA) method [24]. Fifty mg of stem tissue ground in liquid N2 from 3 different plants per cultivar (i.e., 3 biological replicates) was used for lignin content determination.

### DNA microarray

Total RNA from stem sections of both DH and YN were extracted as described in Carpenter and Simon [25] followed by clean-up using the commercial RNeasy mini kit (Qiagen, Valencia, CA, USA). Two biological replicates were collected for each sample. RNA amplification, labeling with cy3- or cy5-dCTP dyes (GE Healthcare, Buckinghamshire, UK), and probe fragmentation was carried out using Ambion AminoAllyl MessageAmp II RNA amplification kit according to the manufacturer’s instructions (Ambion, Austin, TX, USA). A spotted 15 K 50-mer *B. napus* oligo array, previously developed at the Saskatoon Research and Development Centre, Agriculture and Agri-Food Canada [26], was hybridized with the cy5- and cy3-labelled probe pairs. Cy3 dye was used for labeling the YN RNA and Cy5 for DH RNA. A dye swap (cy3/cy5) labelling experiment was performed for each biological replicate. Labeling, hybridization and post- hybridization washing were conducted according to the protocol for Corning epoxide slides (Corning Inc., Lowell, MA, USA). Hybridization was carried out at 37 °C for 17 h in a MAUI hybridization station (BioMicro Systems, Salt Lake City, UT, USA). Following the post-hybridization washes, slides were scanned with a VersArray ChipReader laser scanner (Bio-Rad Laboratories, Hercules, CA, USA).

Image files were transferred to ArrayPro Analyzer software (Media Cybernetics Inc., Bethesda, MD, USA) for image analysis, spot verification, normalization, filtering and feature extractions. A standard statistical program GeneSpring GX (Agilent Technologies, Santa Clara, CA, USA) was used to determine that the data set would fit a normal distribution (log 10) and to check the validity of expression data by assessing the variation between control spots. Data files for the duplicates on individual slides and the dye swap data files for each experiment were merged and average spot intensities used to reduce experimental bias. Normalization was performed on merged data using the Lowess (sub-grid) method, and the local background was subtracted from the values of each spot. The intensity of each spot at λ549 nm (Cy5) and λ647 nm (Cy3) was analyzed using a BASE plug-in and finally transformed into a DH/YN ratio value that denoted the most upper (section 1) and lowest stem sections (section 4), including DH1:YN1, DH4:YN4, DH1:DH4 and YN1:YN4. Additional data processing was performed using tools available in BASE (*http://base.thep.lu.se*). Gene ontology (GO) analysis was conducted using the TAIR database [27]. Microarray ratios with transcript changes > 2-fold upregulated or downregulated between two different *B. napus* stem sections were considered for all ratios where *p* ≤ 0.05 (Additional files 1, 2, 3, 4, 5, 6, 7, 8, 9, 10, 11 and 12: Table S1A and B to S6A and B). Data in these 12 Additional files were screened using either Excel or Microsoft Access to identify individual or groups of *B. napus* genes expressed in multiple DH and YN ratios in the main categories of cell wall, other carbohydrate genes and transcription factors, and in additional subcategories. Ten major transcription factor families, Arabidopsis homologue IDs and corresponding mutant lines for selected *B. napus* genes were retrieved from *http://www.arabidopsis.org/browse/genefamily/index.jsp* [28].

### Quantitative real-time RT-PCR

For quantitative real time reverse transcription PCR (qRT-PCR) experiments, total RNA was extracted from stem sections using a TRIzol reagent (*http://www.invitrogen.com*) following the manufacturer’s instructions. Three biological replicates (independent RNA preparations) were collected and used with three technical reps for qRT-PCR analysis. RNA concentration was determined using a nanodrop spectrophotometer (Fisher Scientific, Canada). Twenty micrograms of total RNA (per sample in each replicate) was treated with Turbo DNase (*www.ambion.com*) as per the manufacturer’s instructions to eliminate trace amounts of genomic DNA. Reverse transcription reactions were performed with Superscript™ III Reverse Transcriptase (*http://**www.invitrogen.com*) according to the manufacturer’s instructions using 1.5 μg RNA per reaction. Two RT reactions per sample were done and reactions were pooled after 10-fold dilution with nuclease-free water. Polymerase chain reactions were carried out using 96-well plates in a LightCycler® 480 II (*http://www.roche-applied-science.com/lightcycler-online*) using SYBR® Green. Reactions contained 10 μl of 2× SYBR® Green Master Mix (Roche), 5 μl of diluted cDNA and 200 nM of gene/EST-specific primer (Additional file 13: Table S7) in a final volume of 20 μl. For data normalization, five reference genes (actin, adenine phosphorybosyl transferase, β-tubulin, cyclophilin and elongation factor 1-α) were included in the experiment and two genes with stable expression, namely β-tubulin and actin, were selected using geNorm software [29]. PCR efficiency, in the range of 85 to 100%, was determined from amplification plots using the program LinRegPCR [30].

### Plant transformation

RNAi and over-expression constructs were developed using either the closest Arabidopsis homologue according to Hossain et al. [31, 32] or the closest alfalfa stem cDNA according to Li et al. [33]. Alfalfa primers were identified by Laberge (personal communication). Floral bud transformation was conducted in Arabidopsis according to Hossain et al. [31, 32]. Alfalfa transformation was conducted using *Agrobacterium tumefaciens* according to Aung et al. [34].

## Results

### DH and YN stems differ in lignin and exhibit different gene expression profiles

**Fig. 1:**
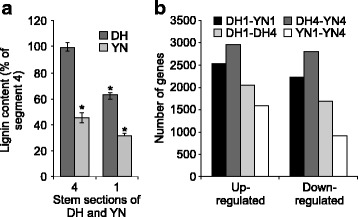
Top and basal section of stem from *B. napus* DH12075 (DH) and YN01–429 (YN) used for lignin analysis. **a** Lignin in top section (1) and basal section (4). **b** Total number of changed gene transcripts in microarray analysis of four pairwise comparisons of the transcriptome in sections 1 and 4. For A, each value is the mean ± SD of 3 biological experiments each replicated 3 times. Values with asterisks varied significantly where *p* ≤ 0.05

To validate our introductory hypothesis, we first determined the stem lignin content of DH and YN plants. Total lignin content showed higher levels in both apical (section 1) (DH1) and basal sections (section 4) (DH4) of DH compared to their counterparts in YN cultivar sections (YN1 and YN4) (Fig. 1a). Within stem sections of each cultivar, we found the highest lignin content to be in the basal sections and the lowest in the apical sections. Increased lignin accumulation is the result of increased enzyme activity and/or increased gene expression. Hence, we compared global gene expression levels between the two cultivars and also between these sections using a 15 K *B. napus* microarray that was used earlier to investigate gene expression in developing *B. napus* seed [35] and diseased stem sections [26]. In detail, we related lignin levels with transcriptome changes between DH and YN (both apical and basal sections for each cultivar (Fig. 1b; Additional files 1, 2, 3, 4, 5, 6, 7, 8, 9, 10, 11 and 12: Tables S1A and B to S6A and B). The greater upregulation of genes in the apical stem sections compared with basal stem section expression pattern is reasonable because the apical region of the plant is developmentally more active compared to the basal region.

### Functional classification of genes up-regulated in high lignin stems of *B. napus*

**Fig. 2:**
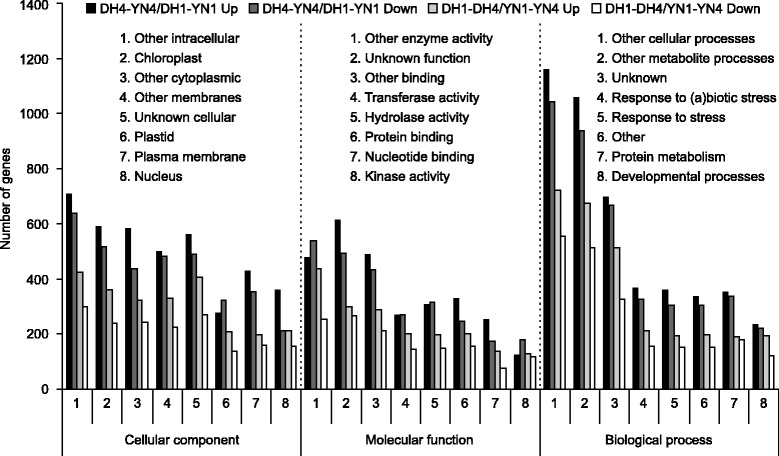
Functional classification of differentially expressed genes in pairwise microarray comparisons of stem section transcriptomes from *B. napus* DH12075 (DH) and YN01–429 (YN). 1, section 1. 4, section 4. Up, upregulated. Down, down regulated. Genes were first compiled into three large biochemical classes and then subdivided into as many as 16 sub-categories. The eight largest sub-categories are shown

Gene annotation of sequences differentially expressed using the 15 K *B. napus* array was conducted using The Arabidopsis Information Resource (*http://www.arabidopsis.org*). Annotated genes were grouped into three major functional categories: cellular component, molecular function and biological process, and then divided into subcategories (Fig. 2; Table 1). For ease of presentation, data was expressed as the average of genes in each subcategory in DH vs. the average in YN. Within the category of biological processes in DH4 vs. YN4, genes involved in defense responses were 7.6% of the total, whereas stress responsive genes were 7.4%; genes involved in developmental processes were 4.9 and 4.7% were involved in transport; 2.35% of genes were involved in transcription and 0.6% in DNA or RNA metabolism. In the cellular component category, 6.4% genes were related to plasma membrane, and nucleus genes accounted for 6.02%; cell wall genes were 2.12 and 1.6% genes were related to cytoplasm functions. In the molecular function category, 7.4% genes were involved in protein binding, and 5.7% genes accounted for DNA or RNA binding activity; 4.8% genes were involved in transporter activity, and 3.4% genes showed transcription factor activity (Fig. 2; Table 1).

**Table 1:** Total number of changed stem transcripts in three major functional categories of *B. napus* DH12075 (DH) and YN01–429 (YN)

	DH4:YN4		DH1:YN1		DH1:DH4		YN1:YN4	
	Up	Down	Up	Down	Up	Down	Up	Down
Cellular Component								
Other intracellular components	754	693	668	580	475	396	377	205
Chloroplast	602	567	581	462	413	320	308	159
Other cytoplasmic components	636	469	531	403	361	321	280	161
Other membranes	531	482	466	483	374	290	284	156
Unknown cellular components	607	607	513	373	463	352	347	187
Plastid	291	343	260	303	174	151	246	122
Plasma membrane	438	381	421	326	262	240	133	79
Nucleus	392	227	331	200	247	203	181	107
Other cellular components	284	285	184	153	225	162	163	103
Mitochondria	198	191	210	188	125	94	97	55
Cell wall	127	123	115	88	75	66	64	40
Cytosol	121	102	106	80	66	66	56	31
Extracellular	110	82	82	62	66	62	44	33
ER	90	76	81	60	60	48	44	33
Golgi apparatus	49	43	40	37	31	26	18	12
Ribosome	23	23	23	18	16	13	10	8
Molecular Function								
Other enzyme activity	515	695	439	383	492	295	383	216
Unknown molecular functions	650	471	578	515	341	376	259	156
Other binding	525	469	455	393	327	274	248	150
Transferase activity	295	308	243	229	211	186	188	100
Hydrolase activity	341	328	275	299	240	199	151	98
Protein binding	351	260	306	230	223	199	176	115
Nucleotide binding	279	132	231	217	131	96	139	53
Kinase activity	137	245	114	110	175	143	85	88
DNA or RNA binding	257	182	160	183	83	125	121	73
Transporter activity	164	201	197	131	153	93	70	56
Other molecular functions	185	157	143	110	122	93	103	45
Transcription factor activity	165	149	130	115	117	93	79	47
Nucleic acid binding	119	111	98	102	76	64	61	28
Structural molecule activity	35	22	28	25	26	16	20	8
Receptor binding or activity	15	18	17	12	16	10	9	5
Biological Process								
Other cellular processes	1254	1145	1066	943	803	710	641	398
Other metabolic processes	1139	1016	974	860	754	664	597	364
Unknown biological processes	763	760	637	575	583	418	444	233
Response to abiotic or biotic stimulus	387	373	350	279	221	197	200	115
Response to stress	381	342	337	263	206	188	181	115
Other biological processes	353	334	325	269	215	197	183	105
Protein metabolism	375	359	333	317	247	229	130	127
Developmental processes	251	261	222	181	197	158	189	83
Transport	266	232	232	193	165	124	139	85
Cell organization and biogenesis	171	154	150	137	127	110	97	56
Transcription	159	146	130	119	104	88	73	42
Electron transport or energy pathways	81	74	61	54	51	31	45	17
Signal transduction	75	52	66	46	39	38	37	22
DNA or RNA metabolism	35	27	24	21	25	20	18	14

*NB* data was ranked in descending order using the DH4-YN4 pair wise comparison. Up., upregulated. Down, down regulated

Annotation for genes differentially expressed in DH1 vs. YN1, DH1 vs. DH4 and YN1 vs. YN4 pairwise comparisons was also carried out. In all three functional categories, genes belonging to the above three groupings showed a similar pattern of distribution to those of DH4 vs. YN4.

### Validation of microarray data by quantitative RT-PCR

**Fig. 3:**
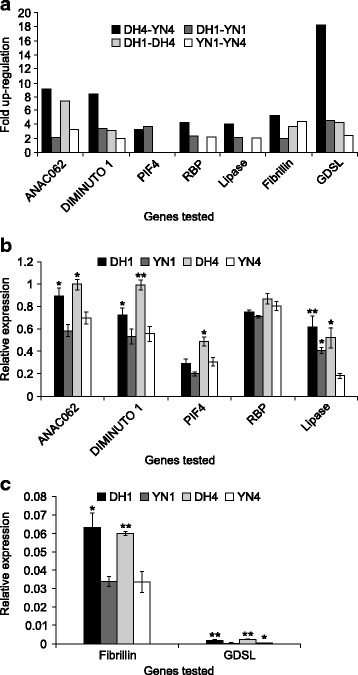
Relative expression pattern of seven regulatory and biochemical genes in stem sections DH12075 (DH) and YN01–429 (YN) using qRT-PCR. **a** Each value is the mean ± SD from 2 biological replicates each technically replicated 3 times. **b** and **c**. Each value is the mean ± SD from 3 biological replicates, each technically replicated 3 times. 1, stem section 1. 4, stem section 4. Values with asterisks represent significantly different data of DH relative to YN where * p ≤ 0.05 and ** *p* ≥ 0.01. The significance test was done separately for each gene

To validate the results of microarray experiments, transcript levels of seven genes were examined by qRT-PCR using total RNA prepared from stem sections. Emphasis was placed on functional diversity in selecting the following genes for qRT-PCR experiments; *ANAC062* (Accession # ES901271), *PHYTOCHOME INTERACTING FACTOR 4* (*PIF4*; EV181356), *GLYCINE-RICH RNA BINDING PROTEIN* (*RBP*/ CAA78513.1), *DIMINUTO 1*, *LIPASE 1* (AY866419), *FIBRILLIN* (AF084554.1) and *GDSL-MOTIF CONTAINING LIPASE/HYDROLASE* (CN730052) (Fig. 3a and b; Additional file 13: Table S7). From the microarray data, it was determined that these selected genes were upregulated in the DH cultivar with high lignin content. The expression profiles of most of the genes tested by qRT-PCR were consistent with our microarray data, although the difference in expression was less in the latter data set. Expression of *DIMINUTO 1* was significantly higher in the basal section of DH (DH4). Expression of *ANAC062* was also higher in the basal section of DH4, whereas *PIF4* showed higher expression in both apical (DH1) and basal sections (DH4). Expression of *LIPASE1* was also higher in both sections of DH compared to those of YN. Transcripts of *RBP* exhibited slight numerical increase in both sections of DH, although the difference was not statistically significant. Because of their extremely low expression levels relative to other tested genes, the two genes encoding *FIBRILLIN* and *GDSL-MOTIF PROTEIN* were assessed separately (Fig. 3c).

Both of the latter genes showed significantly higher expression in the two sections of DH relative to their counterparts in YN.

### Upregulation of monolignol biosynthetic genes

Genes involved in monolignol biosynthesis were retrieved from the TAIR database (*www.arabidopsis.org*) and used to identify *B. napus* biochemical genes that were upregulated at-least 2-fold in high lignin-containing stem material using the microarray data (Table 2). Tissue comparisons showed that several genes encoding enzymes involved in monolignol biosynthesis were upregulated in the DH cultivar relative to the YN cultivar. Among these genes, caffeic acid/5-hydroxyferulic acid *O*-methyltransferase (*OMT1*) was upregulated in the top and basal sections of DH relative to the top and basal sections of YN (Table 2). In addition, *S*-adenosyl methionine synthase (SAMS) and CCoAOMT enzymes that methylate lignin precursors [36] were coded by strongly upregulated genes in our microarray experiments (Table 2). SAMS catalyzes the transfer of an adenosyl group from ATP to the sulphur atom of methionine, resulting in the synthesis of SAM, a common methyl group donor. In the microarray experiments, *SAMS3* was one of the stronger upregulated genes in the top and basal sections of DH relative to those of YN and in the basal section relative to the top section of DH (Table 2).

**Table 2:** Differential upregulation of monolignol biosynthetic genes ≥ two-fold in stem sections of *B. napus* DH12075 (DH) and YN01–429 (YN)

Probe name	Upregulated expression				Corresponding Arabidopsis Loci	Description
	DH4:YN4	DH1:YN1	DH4:DH1	YN4:YN1		
BN18476		2.04		2.13	AT1G09500	Similar to *Eucalyptus gunnii* alcohol dehydrogenase
BN10302	3.64	4.25			AT1G12840	De-etiolated 3 (DET3)
BN21410 BN21411	6.45	2.18		2.25	AT1G15950	Cinnamoyl CoA reductase (CCR1)
BN26995				2.27	AT1G33030	O-methyltransferase family 2 protein
BN17817			2.54		AT1G67980	Caffeoyl-CoA 3-O-methyltransferase
BN24319 BN24320			2.43		AT1G77520	O-methyltransferase family 2 protein
BN23842		2.5			AT1G80820	Cinnamoyl CoA reductase (CCR2)
BN25563		2.14		2.19	AT2G02400	Cinnamoyl-CoA reductase family
BN15586	8.73	2.64			AT2G40890	Coumarate 3-hydroxylase (C3H), a P450-dependent monooxygenase (CYP98A3)
BN26731	2.99				AT2G33600	Cinnamoyl-CoA reductase family
BN14781 BN14782 BN14783	8.79	4.72	2.9		AT3G17390	S-Adenosylmethionine synthetase 3 (SAMS3)
BN22074	4.24				AT3G19450	Cinnamyl-Alcohol Dehydrogenase (CAD4)
BN18533 BN18534	3.09		3.51	2.48	AT4G36220	Ferulic acid 5-hydroxylase 1 (FAH1)
BN13060			2.18		AT5G14700	Cinnamoyl-CoA reductase-related
BN19086 BN19087	3.0			3.26	AT5G48930	Hydroxycinnamoyl-CoA shikimate/quinate hydroxycinnamoyl transferase (HCT)
BN13706	5.22	5.01			AT5G54160	Caffeic acid/5-hydroxyferulic acid O-methyltransferase (OMT1)
BN20498	2.49				AT5G58490	Cinnamoyl-CoA reductase family

Cinnamoyl-CoA reductase (CCR) and cinnamyl alcohol dehydrogenase (CAD) also play important roles in monolignol biosynthesis. The cinnamoyl-CoA esters, precursors of monolignol biosynthesis, are generated by the general phenylpropanoid pathway and then converted into monolignols by CCR and CAD [7]. *CCR1* was among the most upregulated genes in the top and basal sections of DH relative to those of YN, and the same gene was upregulated in the basal section relative to the top section of YN in the array (Table 2). *CCR2* was also upregulated in the top section of DH relative to the top section of YN. Another major gene change in the phenylpropanoid pathway was coded by *p*-coumarate 3-hydroxylase (C3H), also known as *REDUCED EPIDERMAL FLUORESCENCE* (*REF8*) was upregulated in the top and basal sections of DH relative to those of YN (Table 2).

The *DE-ETIOLATED 3* (*DET3), At1g12840,* was upregulated in the top and basal section of DH relative to the top and basal section of YN (Table 2). This gene encodes the C-subunit of the V-type ATPase [37] implicated in the maintenance of pH homeostasis in plants [38]. Moreover, the *FAH1* gene was upregulated in the basal section of DH4 relative to the basal section of YN4, and the same gene is upregulated in the basal section relative to the top section of both cultivars (Table 2). The basal section is more mature and richer in lignin content relative to the top section (Fig. 1a), suggesting the involvement of this gene with lignin accumulation in Brassica. The ferulate-5-hydroxylase (F5H) enzyme is a key regulatory point in the determination of lignin monomer composition [39] by catalyzing the conversion of ferulic acid to sinapic acid and syringyl lignin.

### Differential expression of glucosyltransferase, peroxidase and laccase genes

It is well established that monolignols are synthesized in the cytoplasm and then exported into the cell wall for polymerization [7]. Cellular homeostasis of these monomers is regulated through glucosylation [40]. This modification step increases the solubility and stability of monomers and provides access to the membrane transport systems for transportation and storage. Glucosyltransferases play an important role in these processes [7]. After transport of the monolignols to the cell wall, lignin is formed through dehydrogenative polymerization of the monolignols [7]. The dehydrogenation to monolignol radicals has been attributed to different classes of oxidative proteins, such as peroxidases, laccases, polyphenol oxidases and coniferyl alcohol oxidase. However, it is still unknown which particular peroxidase and laccase genes are directly involved in this process during secondary wall formation.

To shed light on the differential expression pattern of glucosyltransferases, peroxidases and laccases in *B. napus* stem, we retrieved genes that belong to these groups from *www.arabidopsis.org*. A total of 26 glucosyltransferase genes were upregulated 2-fold or more in high lignin containing stem materials (Table 3), whereas 29 and 2 genes belonging to peroxidases and laccases, respectively, were upregulated 2-fold or more (Table 4). Most glucosyltransferases, peroxidases and laccases were upregulated two- to four-fold in more lignified tissues (Tables 3 and 4).

**Table 3:** Differential upregulation of glucosyltransferase genes ≥ two-fold in stem sections of *B. napus* DH12075 (DH) and YN01–429 (YN)

Probe name	DH4:YN4	DH1:YN1	DH4:DH1	YN4:YN1	Corresponding Arabidopsis loci	Description
BN18392				2.44	AT1G05530	UDP-GLUCOSYL TRANSFERASE 75B2
BN25565		2.12			AT1G07250	UDP-GLUCOSYL TRANSFERASE 71C4
BN19757	2.83				AT1G23870	*Arabidopsis thaliana* TREHALOSE-PHOSPHATASE/SYNTHASE 9
BN18181				2.28	AT1G55850	CELLULOSE SYNTHASE LIKE E1
BN20190		5.55	2.19	2.09	AT1G70290	TREHALOSE −6-PHOSPHATASE SYNTHASE S8
BN17018			3.95		AT2G31750	UDP-GLUCOSYL TRANSFERASE 74D1
BN11938		2.24			AT2G31960	GLUCAN SYNTHASE-LIKE 3
BN13391		2.12	2.39		AT2G36850	GLUCAN SYNTHASE-LIKE 8
BN18400			2.05		AT2G43820	UDP-GLUCOSYLTRANSFERASE 74F2
BN19396			2.01		AT3G03050	CELLULOSE SYNTHASE LIKE D3
BN25630	2.35				AT3G50740	UDP-GLUCOSYL TRANSFERASE 72E1
BN16340	2.26				AT3G55830	ECTOPICALLY PARTING CELLS
BN19754			12.95		AT4G01070	UDP-GLUCOSE-DEPENDENT GLUCOSYLTRANSFERASE 72 B1
BN18305		2.23			AT4G02280	SUCROSE SYNTHASE 3
BN17034		2.09			AT4G03550	GLUCAN SYNTHASE-LIKE 5
BN16416		2.33			AT4G10120	ATSPS4F
BN26358	2.56				AT4G15490	UGT84A3
BN25190		2.69			AT4G17770	TREHALOSE −6-PHOSPHATASE SYNTHASE S5
BN20567	3.81	2.87			At4g31590	CELLULOSE-SYNTHASE LIKE C5
BN12852	7.67	2.04			At4g32410	CELLULOSE SYNTHASE 1
BN22038	4.45	2.19			AT4G34135	UDP-GLUCOSYLTRANSFERASE 73B2
BN23469			3.48	2.43	AT4G39350	CELLULOSE SYNTHASE A2
BN20784	2.13				AT5G05170	CELLULOSE SYNTHASE 3
BN26739				2.21	AT5G05870	UDP-GLUCOSYL TRANSFERASE 76C1
BN12647	4.39	2.08			AT5G20830	SUCROSE SYNTHASE 1
BN17387	2.38	2.32			AT5G64740	CELLULOSE SYNTHASE 6

**Table 4:** Differential upregulation of peroxidase and laccase genes ≥ two-fold in stem sections of *B. napus* DH12075 (DH) and YN01–429 (YN)

Probe name	DH4: YN4	DH1:YN1	DH4:DH1	YN4:YN1	Corresponding Arabidopsis loci	Description
*Peroxidase genes*						
BN24392	2.23				AT1G05260	RARE COLD INDUCIBLE GENE 3
BN18476		2.04			AT1G09500	similar to *Eucalyptus gunnii* alcohol dehydrogenase
BN10302		4.25			AT1G12840	ARABIDOPSIS THALIANA VACUOLAR ATP SYNTHASE SUBUNIT C
BN21410		3.73			AT1G15950	CINNAMOYL COA REDUCTASE 1
BN14306	2.16		2.54		AT1G20620	CATALASE 3
BN18084	6.60		2.27		AT1G20630	CATALASE 1
BN16003	2.56				AT1G44970	Peroxidase, putative
BN27576			2.14		AT1G48130	ARABIDOPSIS THALIANA 1-CYSTEINE PEROXIREDOXIN 1
BN11140				2.02	AT1G71695	Peroxidase 12
BN23842		2.50			AT1G80820	CINNAMOYL COA REDUCTASE
BN25563		2.41			AT2G02400	Cinnamoyl-CoA reductase family
BN13322	2.48				AT2G25080	GLUTATHIONE PEROXIDASE 1
BN11700	2.0				AT2G30860	GLUTATHIONE S-TRANSFERASE PHI 9
BN19562	3.68				AT2G38380	Peroxidase 22 (PER22)
BN15586		3.54			AT2G40890	CYP98A3, encodes coumarate 3-hydroxylase (C3H)
BN24228			2.77		AT3G03670	Peroxidase, putative
BN13555	2.09				AT3G11630	Encodes a 2-Cys peroxiredoxin (2-Cys PrxA)
BN14781 82,83,84		4.72			AT3G17390	S-ADENOSYLMETHIONINE SYNTHETASE 3
BN19400	7.76				AT3G49110	PEROXIDASE 33
BN16513	4.57		2.62	2.02	AT4G21960	PEROXIDASE PRXR1
BN24317				2.03	AT4G26010	Peroxidase, putative
BN23955			2.67		AT4G30170	Peroxidase, putative
BN27325 BN13535	4.35		2.17	2.17	AT4G35000	ASCORBATE PEROXIDASE 3
BN16234	4.13				AT4G36430	Peroxidase, putative
BN22581	3.69				AT4G37530	Peroxidase, putative
BN14948	3.30				AT5G06290	2-CYSTEINE PEROXIREDOXIN B
BN19087		2.29			AT5G48930	HYDROXYCINNAMOYL-COA SHIKIMATE/QUINATE HYDROXYCINNAMOYL TRANSFERASE
BN13706		5.01			AT5G54160	O-METHYLTRANSFERASE 1
*Laccase genes*						
BN23268			2.17		AT2G38080	LACCASE-LIKE MULTICOPPER OXIDASE 4
BN14150	3.23				AT5G60020	LACCASE 17

### Genes involved in carbohydrate biosynthesis

The importance of cellulose and its integration with lignin and pectin in plant cell walls led us to determine transcript levels for genes coding for cellulose biosynthetic enzymes in the microarray experiment. Genes involved in cellulose biosynthesis in Arabidopsis were downloaded from *www.arabidopsis.org* and were compared with our microarray data to identify genes that were upregulated by 2-fold or more in high lignin-containing material (Table 5, Additional file 14: Table S8). Comparisons showed that several genes encoding enzymes involved in cellulose biosynthesis were upregulated in the basal section of the cultivars. *CELLULOSE SYNTHASE A1* (*BnCESA1*; BN12852) was strongly upregulated in the basal section of DH relative to its counterpart in YN (Table 5). *CESA2* (BN17389) was also upregulated in the basal section of both cultivars relative to the top sections, while *CESA6* was upregulated in the top and basal sections of DH relative to the top and basal sections of YN in the array experiments. *DEFECTIVE GLYCOSYLATION* (*DGL1*) (BN19297) was upregulated in the top and basal sections of DH relative to the top and basal sections of YN (Table 5). *DGL1* encodes a plant ortholog of human SOT48 or yeast WBP1 and is an essential protein subunit of the oligosaccharyltransferase (OST) complex.

**Table 5:** Differential upregulation of carbohydrate biosynthetic genes by ≥2-fold in stem sections of *B. napus* DH12075 (DH) and YN01–429 (YN)

Probe name	Upregulated expression				Corresponding Arabidopsis Loci	Description
	DH4: YN4	DH1: YN1	DH4: DH1	YN4: YN1		
BN18181				2.28	AT1G55850	CELLULOSE SYNTHASE LIKE E1
BN17967		3.43			AT1G71100	RADIAL SWELLING 10 (RSW10)
BN12852	7.67				AT4G32410	CELLULOSE SYNTHASE 1 (CESA1)
BN23469			3.48	2.43	AT4G39350	CELLULOSE SYNTHASE A2 (CESA2)
BN20787	2.44				AT5g05170	Constitutive Expression of VSP1 (CEV1)
BN18701 BN18702	6.43		2.21		AT5G19220	ADP GLUCOSE PYROPHOSPHORYLASE LARGE SUBUNIT 1 (APL1)
BN19160	2.66	4.97	2.21		AT5G49720	ARABIDOPSIS THALIANA GLYCOSYL HYDROLASE 9A1 (ATGH9A1)
BN17389	3.1	2.22			AT5G64740	CELLULOSE SYNTHASE 6 (CESA6)
BN19297	8.49	5.27			AT5G66680	DEFECTIVE GLYCOSYLATION (DGL1)

Other genes involved in carbohydrate synthesis were also upregulated in DH stems (Table 5). One of these, *RADIAL SWELLING 10* (*RSW10*) (BN17967), was upregulated in the top section of DH relative to the top section of YN (Table 5). RSW 10 encodes a ribose 5-phosphate isomerase involved in the formation of uridine used for the synthesis of UDP-sugars.

Plant primary carbohydrate metabolism is complex, yet flexible, and is regulated at many levels. To gain molecular insights into the carbohydrate metabolic process in *B. napus* stem, we retrieved genes involved in cellular carbohydrate metabolic process from *www.arabidopsis.org*. A total of 419 loci were retrieved and compared to identify upregulated genes in the arrays. In total, 85 genes were found to be upregulated of which 52, 33, 35 and 29 belonged to DH4:YN4, DH1:YN1, DH4:DH1 and YN4:YN1, respectively (Additional file 14: Table S8).

### Targets for transcriptional regulation of cell wall formation

Transcriptional regulation plays a key role in the complex series of events leading to cell wall formation [41]. To identify transcription factors (TFs) that were upregulated in the array, a search of our data was performed using 10 major transcription factors from the *A. thaliana* Transcription Factor Database [42]. The search revealed that a wide range of TFs were upregulated and downregulated in DH vs. YN stems. Several gene families, zinc finger (C2H2 and C3HC4), basic/helix-loop-helix (bHLH), basic region/leucine zipper motif (bZIP), AP2/EREBP and NAC (NAM/ATAF1,2/CUC2), were over-represented among the differentiated TFs (Table 6; Additional files 1, 2, 3, 4, 5, 6, 7, 8, 9, 10, 11 and 12: Tables S1A and B to S6A and B).

**Table 6:** Ten major transcription factors differentially expressed 2-fold or more in stem sections of *B. napus* DH12075 (DH) and YN01–429 (YN)

Transcription factor	Sample groups in microarray							
	DH4-YN4		DH1-YN1		DH1-DH4		YN1-YN4	
	Up	Down	Up	Down	Up	Down	Up	Down
C2H2	16	22	14	11	4	7	6	5
C3HC4	13	15	12	9	3	4	2	2
bHLH	13	11	11	7	7	3	3	1
bZIP	9	15	4	7	6	5	7	4
NAC	8	7	8	4	6	2	4	1
AP2-EREBP	8	8	4	6	4	3	1	1
Homeobox	6	8	6	3	4	4	3	4
MYB	4	2	3	0	4	4	5	2
MADS	4	4	1	3	3	3	3	3
WRKY	4	6	2	5	2	3	2	1

The largest group of transcription factors with differential expression (25 gene loci; 13 upregulated and 12 downregulated) consisted of Cys2His2-like (C2H2) zinc finger genes. C2H2 zinc fingers are well known and display a wide range of functions, from DNA and RNA binding to the involvement in protein-protein interactions. In our array data, the numbers of differentially expressed members (> 2-fold) for this family in DH4:YN4, DH1:YN1, DH1:DH4 and YN1:YN4 were 38, 25, 11 and 11, respectively (Table 6; Additional files 1, 2, 3, 4, 5, 6, 7, 8, 9, 10, 11 and 12: Tables S1A and B to S6A and B). BN21811 related to *At1g14580*, a member of this family, was over-expressed in all 4 groups whereas the other 3 family members were upregulated in 3 groups. Another gene, BN15654 related to *At5g04240*, was upregulated in the basal section of DH relative to the basal section of YN. This latter gene is a member of the jumonji group of C2H2 transcription factors (Table 6; Additional files 1, 2, 3, 4, 5, 6, 7, 8, 9, 10, 11 and 12: Tables S1A and B to S6A and B).

C3HC4 zinc finger genes formed the next largest gene category (Table 6). One of these genes BN22477 (related to At5g05830) was strongly upregulated in three of the four tissue comparisons and another in two comparisons. In contrast, BN17974 (related to At3g09770) was strongly down regulated in the DH4:YN4 and DH4:DH1 comparisons. B helix loop helix transcription factors were the third most differentially expressed genes in DH1:YN1, DH1:DH4 and YN1:YN4, but to a lesser extent than in DH4:YN4 (Table 6). In more detail, BN155636 related to *At2g18300* (AtbHLH64) and BN12702 related to *At1g05805* (AtbHLH128) were over-expressed in three groups of our array (Additional files 1, 2, 3, 4, 5, 6, 7, 8, 9, 10, 11 and 12: Tables S1A and B to S6A and B). AtbHLH64 is known to play a role in Arabidopsis cytokinin signaling [43]. Though the role of bHLH TFs in lignin regulation is correlative rather than firmly established, we cannot rule out their possible involvement considering the common precursors of the lignin and flavonoid pathways and the known influence of bHLH on the flavonoid pathway genes, as well as the known interaction between bHLHs and MYB TFs in other physiological and developmental systems such as Brassica trichome development [44].

Plant basic region/leucine zipper motif (bZIP) transcription factors regulate processes including pathogen defense, light and stress signaling, seed maturation and flower development in Arabidopsis [45]. The numbers of bZIP family members differentially expressed more than 2-fold in DH4-YN4, DH1-YN1, DH1-DH4 and YN1-YN4 were 24, 11, 11 and 11, respectively in the array data (Table 6). BN15088 related to *At2g18160* (ATBZIP2) were upregulated in 3 groups.

AP2 (APETALA2) and EREBPs (ethylene responsive element binding proteins) are a family of transcription factors unique to plants and share a highly conserved region of about 60 to 70 amino acids (the so-called AP2 domain) with no apparent similarity outside this domain. The numbers of differentially expressed members (> 2-fold) for this family in DH4-YN4, DH1-YN1, DH1-DH4 and YN1-YN4 were 16, 10, 7 and 2, respectively, in the array data (Table 6). In detail, BN16694 related to *At1g68550* (*AtERF118*), was upregulated in 3 groups in our microarray experiments. Another of these genes, BN16341 related to *At5g67190* (DREB2) was upregulated in the basal section relative to the top section of DH relative to YN. DREB2 contains the conserved DNA binding domain found in ERF (ethylene response factor) and AP2 (APETALA2).

NAC proteins constitute one of the largest families of plant-specific transcription factors, and are expressed in various developmental stages and tissues [46]. The NAC domain was originally characterized from consensus sequences from petunia **N**AM and from Arabidopsis **A**TAF1, ATAF2 and **C**UC2. In our array data, the number of differentially expressed members (> 2-fold) for this family in DH4-YN4, DH1-YN1, DH1-DH4 and YN1-YN4 were 15, 12, 8 and 5, respectively (Table 6). One member of this family, BN13387 related to *At1g61110*, was over-expressed in all 4 groups of our array and was previously reported to be expressed in stamens [47]. Other NAC transcription factors were only upregulated in 3 groups (Table 6). BN24136 was highly upregulated and is related to *At3g49530* (ANAC062), a gene that plays an integrative role in plant responses to stress [48]. BN17366 related to *At1g01720* is another Arabidopsis NAC-domain containing protein 1 (ATAF1) upregulated in the basal section relative to the top section of DH stems.

In addition to the six classes of over-represented transcription factors, MYB factors were more than 2-fold upregulated in our microarray comparisons of lignified and less lignified tissues. MYB factors represent a family of proteins with a conserved R2R3 (stimulating) or R3 (inhibiting) MYB DNA-binding domain. They often bind to bHLHs. In our array data, the numbers of overexpressed members of this family in DH4-YN4, DH1-YN1, DH1-DH4 and YN1-YN4 were 6, 3, 8 and 7, respectively (Table 6). BN17384 related to *At5g60890* (AtMYB34) was expressed in all 4 groups of the array data while 2 other members of this family were upregulated in 3 groups (Table 6). BN26939 related to *At2g47460* (AtMYB12) was also strongly upregulated in the array data.

In order to highlight the promising genes for future functional characterization from these elaborated sets, 25 TFs with representation from families mentioned above, plus six biochemical or physiological genes, were selected and their Arabidopsis knockdown mutants retrieved from TAIR (Additional files 14, 15, 16 and 17: Tables S8 to S11). In our analysis on a subsample of these lines, 4 TF lines and 4 non-TF lines showed lower lignin content than the Col-0 control line, while 2 TF lines and 3 non-TF lines showed enhanced lignin content (Additional files 16 and 17: Tables S10 and 11).

## Discussion

The yellow seeded *B. napus* YN01–429 cultivar was recently developed through classical breeding, and was found to contain significantly reduced seed lignin content relative to seeds of the more conventional brown seeded cultivar DH12075 [20, 21]. Here, we determined that the stems of the brown-seeded cultivar were more lignified than those of the yellow-seeded cultivar and that basal stem sections were more lignified than apical stem sections for both cultivars.

### Biochemical genes

Biochemical genes specifying monolignol formation, such as *COMT* [10], *CCoAOMT 1* [49], *SAMS* [50], *CAD* [51], *REF8* (*C3H*) [52] and *F5H* (*FAH5*) [53], were more highly expressed in the more lignified stem tissues we examined. Upregulation in more lignified tissue also occurred for genes specifying monolignol glucosylation, and monolignol polymerization, including peroxidases and laccases, when pairs of tissue sections were compared. Cellulose biosynthesis, the entire cell wall and plant development appeared to be affected by some of these differentially expressed Brassica genes. For example, we found that Brassica *CES* genes [54, 55] and peroxidases [56] were also more upregulated in more lignified tissues. Passardi et al. [56] showed that two highly homologous Arabidopsis peroxidases, AtPrx33 and Atprx34, were involved in cell elongation, and the knockout mutant, *cesa2*, also had severe defects in cell wall formation and microtubule orientation [57]. However, the numbers of cellulose biosynthetic genes upregulated in DH were fewer than lignin biosynthetic genes. Hence, our comparative transcriptome analyses of lignified and less lignified material could serve as a source of differentially expressed biochemical genes that impact on cell wall recalcitrance and could be useful to enable plant molecular breeders to fine tune crop residue composition for industrial applications.

### Targets for transcription regulation

Microarray analysis of differentially lignified tissues resulted in six transcription factor families being more prominent in expression changes than other major TFs. Transcription factors have recently received attention from plant molecular breeders because of their ability to modify entire biochemical pathways or developmental families of genes. For example, overexpression of the Arabidopsis *GLABRA3* (*AtGL3*) *bHLH* transcription factor in *B. napus* enhanced trichome coverage and insect resistance [44, 58].

Here, we screened our microarray data for the top 10 TF families present in lignified *B. napus* stems with the intent of beginning the process of characterizing the function of a subset of regulatory genes using Arabidopsis knockdown mutants. The most modified TF family in our stem microarrays was the zinc finger family. Several zinc finger proteins (ZFPs) in plants, e.g. Arabidopsis and petunia, have already been found to be involved in a variety of processes such as the regulation of floral organogenesis, leaf initiation, lateral shoot initiation, gametogenesis and stress response [59]. The C2H2 group was the most highly upregulated set of zinc finger families in our analyses. This latter group is known to be involved in the regulation of Arabidopsis flowering time [60]. Prigge and Wagner [61] reported that *SERRATE* (*At2g27100*), another member of the C2H2 family, plays a role in embryogenesis and is transcribed in shoot meristem and in emerging organ primordia throughout development. Dong and co-workers [62] also showed that *SERRATE* is required for the accurate processing of microRNA (miRNA) precursors in the plant cell nucleus. Moreover, some zinc finger genes highly expressed in trichomes have been reported to play a role in cell wall biosynthesis [63]. Thus, selections from among these differentially expressed zinc finger genes could target *B. napus* regulatory genes that can modify entire developmental programs.

BHLH TFs are one of the largest TF families in Arabidopsis. Phylogenetic analysis, divergence in binding site specificity and their varied ability to engage in homodimerization and heterodimerization events strongly support their roles in a multiplicity of transcriptional programs [64, 65]. BHLH TFs are known to play important roles in the flavonoid branch of the phenylpropanoid pathway; hence it makes sense that we would find them so plentiful in the closely related lignified tissue. In Arabidopsis seeds, the MYB transcription factor *TRANSPARENT TESTA 2* (*TT2*) forms a complex together with the bHLH transcription factor *TT8* and a WD40 scaffold protein to control the expression of *BANYULS*, a proanthocyanidin biosynthetic gene [66]. In a leaf study, *TT8* and its closest homolog *GL3* and *ENHANCED GLABRA* (*EGL3*) regulate flavonoid biosynthesis through interaction with two homologous MYB proteins *PRODUCTION OF ANTHOCYANIN PIGMENTS 1* (*PAP1,* MYB75) and *PAP2* (MYB90) [67]. In our stem microarrays, several bHLH genes were also differentially expressed along with a number of *MYB* genes. These genes should be paired and tested to determine whether any are binding partners and whether their modification affects lignin in transgenic plants or mutants. Already, the alfalfa bHLH *TT8* homologue has been shown in RNAi studies to downregulate carbohydrate and dry matter accumulation, but not lignin, in alfalfa forage [68].

*AP2/EREBP* genes represent the fifth most differentially expressed genes in the *B. napus* stem microarrays. These genes play a variety of roles throughout the plant life cycle; from being key regulators of several developmental processes, like floral organ identity determination or control of leaf epidermal cell identity, to forming part of the mechanisms used by plants to respond to various types of biotic and environmental stresses [69]. NAC genes ranked sixth among our most differentially expressed TFs in the stem microarrays. Mitsuda et al. [70] showed that *NAC SECONDARY WALL THICKENING PROMOTING FACTOR1* (*NST1*) and *NST3* are key regulators of the formation of secondary walls in woody tissues of Arabidopsis. Lu et al. [71] showed that *ATAF1* mRNA expression is strongly induced by dehydration and abscisic acid (ABA) treatment and they suggested a general role of this protein as a repressor in drought stress responses. Wu et al. [72] also implicated *ATAF1* in diverse biotic and abiotic stress responses including drought, high-salinity, ABA, methyl jasmonate, mechanical wounding and *Botrytis cinerea* infection. Considering the expression pattern of *NAC* transcription factors in the array data and their role in lignin biosynthesis as well as in diverse biotic and abiotic stress responses, this family is a promising target for lignin modulation of the plant cell wall.

Although not among the most differentially expressed *B. napus* stem TFs, *MYB*-related transcription factors are an important group since they regulate different branches of secondary metabolism, in addition to the identity and fate of plant cells [73]. Studies on *MYB* transcription factors in Arabidopsis, pine and eucalyptus confirmed regulatory roles that *MYB* TFs play in lignin biosynthesis [14, 15, 74, 75]. *MYB58* and *MYB63* are transcriptional regulators specifically activating lignin biosynthetic genes during secondary wall formation in Arabidopsis [75]. Dominant repression of their functions led to a reduction in secondary wall thickening and lignin content, while overexpression of *MYB58* and *MYB63* resulted in specific activation of lignin biosynthetic genes and concomitant ectopic deposition of lignin in cells that are normally unlignified [75]. Newman and co-workers [13] suggested a role for another Arabidopsis *MYB* gene, *AtMYB61*, in cell wall deposition in normal vascular development [76]. However, transcription of these three *MYB* genes was unaltered in our array experiments. Instead, *MYB12* and *MYB34* were differentially regulated in the arrays. *MYB34* plays a key role in the regulation of indole glucosinolate homeostasis in Arabidopsis [77], and *MYB12* acts as a flavonol-specific activator of flavonoid biosynthesis [78]. Luo et al. [79] showed that *MYB12* can also activate the caffeoyl quinic acid **(**CQA) biosynthetic pathway when expressed in a tissue-specific manner in tomato. The role of these two MYB TFs in secondary metabolism pathways and the correlation of their expression patterns with stem lignification establish this family as an important target in the modulation of lignin biosynthesis.

Analysis of Arabidopsis mutants for lignin, eight mutant lines showed lower lignin content than the Col-0 control line, while five showed enhanced lignin content. These verified lignin genes can now be used to modify Brassica lignin using our microarray oligonucleotides. Moreover, the Brassica and Arabidopsis sequences can be used to find homologues in other species. In fact, *DIM1* and *eEF-1Bβ1* have already been shown to modify lignin and carbohydrate in Arabidopsis [31, 32] and *TT8* and *HB12* have been shown to modify carbohydrate structure in alfalfa [34, 68]. In addition, several other genes (*HB5, DIM1, ZFP2, eEF-1Bβ1, ANAC62*) have also been used to modify alfalfa (Amyot, personal communication).

## Conclusions

Most of the structural genes in the lignin biosynthetic pathway in plants have been characterized; however, more investigation is needed in the areas of understanding transcriptional regulation and unraveling the mechanism of monolignol polymerization. Here, we examined the expression pattern of genes in stem sections of two Brassica cultivars with differential lignin content. In doing so, we identified and established expression pattern of many new genes likely involved in cell wall biosynthesis and regulation. This transcript profiling allowed for the identification of novel gene candidates with strong potential at playing key roles in cell wall construction during stem development. The genes represent valuable tools to make cell walls more amenable to hydrolysis. More important, we were able to identify the 10 most important transcription factor families and listed 25 promising TF genes for future characterization. Elucidation of their precise mechanisms of transcriptional activation or repression of the cell wall biosynthetic process will not only shed light on the effect of these transcriptional regulators in several plant species, but may also make it possible for the molecular breeding community to modify entire biosynthetic pathways of cellulose or lignin by altering one or a few transcription factors.

## Additional files

Additional file 1: **Table S1A.** Differential expression of DH1 vs DH4 less than point 5 (microarray). (XLSX 360 kb)

Additional file 2: **Table S1B.** Differential expression of DH1 vs DH4 greater than 2 (microarray). (XLSX 442 kb)

Additional file 3: **Table S2A.** Differential expression of DH1 vs YN1 less than point 5 (microarray). (XLSX 496 kb)

Additional file 4: **Table S2B.** Differential expression of DH1 vs YN1 greater than 2 (microarray). (XLSX 565 kb)

Additional file 5: **Table S3A.** Differential expression of YN1 vs YN4 less than point 5 (microarray). (XLSX 226 kb)

Additional file 6: **Table S3B.** Differential expression of YN1 vs YN4 greater than 2 (microarray). (XLSX 417 kb)

Additional file 7: **Table S4A.** Differential expression of DH4 vs YN4 less than point 5 (microarray). (XLSX 728 kb)

Additional file 8: **Table S4B.** Differential expression of DH4 vs YN4 greater than 2 (microarray). (XLSX 718 kb)

Additional file 9: **Table S5A.** Differential expression of DH2 vs YN2 less than point 5 (microarray). (XLSX 567 kb)

Additional file 10: **Table S5B.** Differential expression of DH2 vs YN2 greater than 2 (microarray). (XLSX 585 kb)

Additional file 11: **Table S6A.** Differential expression of DH3 vs YN3 less than point 5 (microarray). (XLSX 512 kb)

Additional file 12: **Table S6B.** Differential expression of DH3 vs YN3 greater than 2 (microarray). (XLSX 556 kb)

Additional file 13: **Table S7.** qRT-Primers used in this study. (DOC 47 kb)

Additional file 14: **Table S8.** Differential upregulation of genes involved in cellular carbohydrate metabolic processing. (DOC 143 kb)

Additional file 15: **Table S9.** Ten categories of TFs with BN oligonucleotides retrieved from stem section comparisons of *B. napus* DH12075 (DH) and YN01–429 (YN). (XLSX 100 kb)

Additional file 16: **Table S10.** Targeted transcription factors selected for lignin validation using Arabidopsis mutants. (DOCX 25 kb)

Additional file 17: **Table S11.** Selected non-TF genes for lignin validation using Arabidopsis mutants. (DOCX 17 kb)

## Abbreviations

ABA: Abscisic acid
ADP-Glc PPase: ADP-glucose pyrophosphorylase
AP2: APETALA2
bHLH: Basic/helix-loop-helix
bZIP: Basic region/leucine zipper
C3H: *p*-coumarate 3-hydroxylase
CAD: Cinnamyl alcohol dehydrogenase
CCoAOMT: Caffeoyl-coenzyme A *O*-methyltransferase
CCR: Cinnamoyl-CoA reductase
cDNA: Complementary DNA
CESA: CELLULOSE SYNTHASE
DET3: DE-ETIOLATED 3
DGL: DEFECTIVE GLYCOSYLATION
DIM1: DIMINUTO 1
DNA: Deoxyribonucleic acid
EGL: ENHANCED GLABRA
EREBP: Ethylene responsive element binding protein;
F5H: Ferulate-5-hydroxylase
GO: Gene ontology
miRNA: microRNA
NST: NAC SECONDARY WALL THICKENING PROMOTING FACTOR
OMT1: Caffeic acid/5-hydroxyferulic acid *O*-methyltransferase
OST: Oligosaccharyltransferase
PAP: PRODUCTION OF ANTHOCYANIN PIGMENTS
qRT-PCR: Quantitative real time reverse transcription PCR
REF: REDUCED EPIDERMAL FLUORESCENCE
RNA: Ribonucleic acid
RSW: RADIAL SWELLING
SAMS: *S*-adenosyl methionine synthase
TAIR: The Arabidopsis Information Resource
TF: Transcription factor
TGA: Thioglycolic acid
TT: TRANSPARENT TESTA
ZFP: Zinc finger protein
