# Supplementary material for: Transcriptome profiling of Brassica napus stem sections in relation to differences in lignin content
Source: BMC Genomics. 2018 Apr 16;19:255. doi: 10.1186/s12864-018-4645-6 (PMC5903004; doi:10.1186/s12864-018-4645-6)
Supplement: Supplementary file 13 — Table S7. qRT-Primers used in this study. (DOC 47 kb) [file 12864_2018_4645_MOESM13_ESM.doc]

Table 1: List of gene specific primers used for qRT-PCR

| Primer name | Primer sequence | Gene amplified |
| --- | --- | --- |
| ES901271_F | GAACCTGCTGTCTCGTCTC | ES901271 (ANAC062) |
| ES901271_R | CGGTTGTAGCCTCATCAC | ES901271 (ANAC062) |
| EV181356_F | GCAAAGCCCGGTACTGTTGC | EV181356 (PIF4) |
| EV181356_R | GTGCCCTGCATACGTGGGAA | EV181356 (PIF4) |
| AY866419_F | GCGAAATGGAGGTGTATCC | AY866419 (DIMINUTO 1) |
| AY866419_R | GGTGCGTAGTAGACTCCAAC | AY866419 (DIMINUTO 1) |
| AF084554.1_F | GTCCAAGACACGGCTTCTTC | AF084554.1 (fibrillin) |
| AF084554.1_R | ACAAAGACGCTTCCACCATC | AF084554.1 (fibrillin) |
| AY866419_F | GATCGGATGCGCAGTAATCT | AY866419 (Lipase 1) |
| AY866419_R | TCCGGAGCTTGTTGAGTTC | AY866419 (Lipase 1) |
| CAA78513.1_F | GCGAAGTTATCGATTCAAAGATCA | Glycine-rich RNA binding protein (RBP) (CAA78513.1) |
| CAA78513.1_R | CGTTGACGGTGATGGTACG | Glycine-rich RNA binding protein (RBP) (CAA78513.1) |
| CN730052_F | AGTTCCGGCTATGTTTGTGC | GDSL-motif containing lipase/hydrolase (CN730052) |
| CN730052_R | GTTCATGCCGTTGCTAAACC | GDSL-motif containing lipase/hydrolase (CN730052) |
| β-tubulin_F | ATTCGTTGGGAGGAGGAACT | β-tubulin |
| β-tubulin_R | CACAGAGAGCGTTGCATTG | β-tubulin |
| EF1α_F | CTGTTGCTGTCGGTGTCATC | Elongation factor 1-α |
| EF1α_R | AAGCGACCAAACACCAAAC | Elongation factor 1-α |
| APT_F | CCTTCTTGTGCGAGGGAAAT | Adenine phosphorybosyl transferase |
| APT_R | CGCAGCAAACATCGTCTC | Adenine phosphorybosyl transferase |
| Cyclophilin_F | CAGGTGGTGGAAGGGTTAG | Cyclophilin |
| Cyclophilin_R | TGAAGACCGAACACCACTC | Cyclophilin |
| AF111812_F | TTCAATGTCCCTGCCATGTA | Actin (AF111812) |
| AF111812_R | GAGACGGAGGATAGCGTGAG | Actin (AF111812) |
