# Supplementary material for: Transcriptome profiling of Brassica napus stem sections in relation to differences in lignin content
Source: BMC Genomics. 2018 Apr 16;19:255. doi: 10.1186/s12864-018-4645-6 (PMC5903004; doi:10.1186/s12864-018-4645-6)
Supplement: Supplementary file 14 — Table S8. Differential upregulation of genes involved in cellular carbohydrate metabolic processing. (DOC 143 kb) [file 12864_2018_4645_MOESM14_ESM.doc]

Supplementary Table S8: Differential up-regulation of genes involved in carbohydrate metabolic processing in stem sections of DH12075 and YN01-429

| Probe name | Fold up-regulation | | | | Corresponding Arabidopsis locus | Description |
| --- | --- | --- | --- | --- | --- | --- |
|  | DH4-YN4 | DH1-YN1 | DH4-DH1 | YN4-YN1 |  |  |
| BN19040, 41 | 6.47 | 3.59 | 2.05 | 2.37 | AT5G20950 | glycosyl hydrolase family 3 protein |
| BN18325 |  | 2.49 | 2.11 |  | AT1G15380 | lactoylglutathione lyase family protein / glyoxalase I family protein |
| BN26328 | 2.22 | 2.51 |  |  | AT2G14620 | xyloglucan:xyloglucosyl transferase, putative / xyloglucan endotransglycosylase, putative / endo-xyloglucan transferase, putative |
| BN20406, 07 |  | 2.13 | 3.23 |  | AT5G13980 | glycosyl hydrolase family 38 protein |
| BN12089 |  | 4.22 | 2.03 |  | AT3G57790 | glycoside hydrolase family 28 protein / polygalacturonase (pectinase) family protein |
| BN20090 |  | 2.24 | 3.44 | 2.12 | AT3G26380 | glycosyl hydrolase family protein 27 |
| BN24587 | 3.25 | 2.62 |  |  | AT2G16230 | catalytic/ cation binding / hydrolase, hydrolyzing O-glycosyl compounds |
| BN25784 |  | 3.23 |  | 3.53 | AT4G19750 | glycosyl hydrolase family 18 protein |
| BN21071 |  | 3.31 |  |  | AT1G23190 | phosphoglucomutase, cytoplasmic, putative / glucose phosphomutase, putative |
| BN13092 | 3.93 | 3.73 | 2.15 | 2.22 | AT3G55430 | glycosyl hydrolase family 17 protein / beta-1,3-glucanase, putative |
| BN16591 | 2.25 | 5.34 | 2.21 | 2.63 | AT5G04885 | glycosyl hydrolase family 3 protein |
| BN24150 | 2.35 | 2.86 | 2.45 | 2.45 | AT4G23500 | glycoside hydrolase family 28 protein / polygalacturonase (pectinase) family protein |
| BN12307 |  | 4.16 |  | 2.01 | AT3G07840 | polygalacturonase, putative / pectinase, putative |
| BN19266 | 9.86 | 5.94 |  | 2.34 | AT5G56720 | malate dehydrogenase, cytosolic, putative |
| BN23564 |  | 2.51 |  |  | AT3G54440 | glycoside hydrolase family 2 protein |
| BN15349 |  | 3.02 | 2.02 |  | AT5G58330 | malate dehydrogenase (NADP), chloroplast, putative |
| BN11996 | 3.50 | 3.05 | 2.18 | 2.86 | AT5G15140 | aldose 1-epimerase family protein |
| BN25913, 14 | 2.94 | 2.25 | 2.21 | 2.21 | AT3G07320 | glycosyl hydrolase family 17 protein |
| BN12241 |  | 2.11 |  |  | AT3G62710 | glycosyl hydrolase family 3 protein |
| BN19127 |  | 2.53 |  |  | AT4G30310 | ribitol kinase, putative |
| BN23257 | 2.02 | 2.36 |  |  | AT1G19170 | glycoside hydrolase family 28 protein |
| BN14673 |  | 2.39 |  |  | AT5G08380 | Arabidopsis thaliana ALPHA-GALACTOSIDASE 1 (AtAGAL1) |
| BN12936 | 2.43 | 2.05 | 2.27 |  | AT3G43860 | Arabidopsis thaliana Glycosyl Hydrolase 9A4 (AtGH9A4) |
| BN24262 |  | 3.86 |  | 2.28 | AT4G11050 | Arabidopsis thaliana glycosyl hydrolase 9C3 (AtGH9C3) |
| BN21877 |  | 3.0 | 2.32 | 2.27 | AT2G32860 | BETA GLUCOSIDASE 33 (BGLU33) |
| BN24082 |  | 2.41 |  | 2.17 | AT5G36890 | BETA GLUCOSIDASE 42 (BGLU42) |
| BN22669 | 3.79 | 3.81 |  |  | AT1G02640 | BETA-XYLOSIDASE 2 (BXL2) |
| BN16895 | 2.23 | 2.10 |  |  | AT3G13750 | Beta galactosidase 1 (BGAL1) |
| BN27597  BN16462 | 3.56 | 4.12 | 2.26 | 2.04 | AT5G61410 | D-RIBULOSE-5-PHOSPHATE-3-EPIMERASE (RPE) |
| BN21889 | 2.67 | 4.10 |  |  | AT5G17520 | ROOT CAP 1 (RCP1) |
| BN25325 | 4.94 | 5.76 |  |  | AT3G52840 | beta-galactosidase 2 (BGAL2) |
| BN14844 | 3.34 | 4.56 | 2.68 |  | AT5G56870 | beta-galactosidase 4 (BGAL4) |
| BN16716 | 3.68 | 5.89 |  |  | AT1G80460 | nonhost resistance to P. s. phaseolicola 1 (NHO1); Encodes a protein similar to glycerol kinase |
| BN22236 |  |  | 2.16 |  | AT4G37800 | xyloglucan:xyloglucosyl transferase, putative |
| BN18121 |  |  | 3.47 |  | AT3G42950 | glycoside hydrolase family 28 protein / polygalacturonase (pectinase) family protein |
| BN16265 |  |  | 2.27 | 2.06 | AT3G55780 | glycosyl hydrolase family 17 protein |
| BN21265 | 2.53 |  | 2.98 | 3.72 | AT2G20680 | glycosyl hydrolase family 5 protein |
| BN12454 | 3.45 |  | 2.10 |  | AT3G14040 | exopolygalacturonase / galacturan 1,4-alpha-galacturonidase / pectinase |
| BN22502, 03 |  |  | 3.20 | 2.02 | AT5G58090 | glycosyl hydrolase family 17 protein |
| BN24828 |  |  | 4.30 | 2.22 | AT4G34480 | catalytic/ cation binding / hydrolase, hydrolyzing O-glycosyl compounds |
| BN22729 | 4.90 |  | 3.22 |  | AT3G47000 | glycosyl hydrolase family 3 protein |
| BN22961 |  |  | 2.40 |  | AT5G57330 | aldose 1-epimerase family protein |
| BN16933 | 2.55 |  | 2.05 |  | AT1G75940 | encodes a protein similar to the BGL4 beta-glucosidase from Brassica napus/ ATA27 |
| BN13604 |  |  | 2.77 | 2.29 | AT5G24420 | glucosamine/galactosamine-6-phosphate isomerase-related |
| BN24082 |  |  | 2.70 |  | AT5G36890 | BETA GLUCOSIDASE 42 (BGLU42) |
| BN13150 | 2.17 |  | 2.25 |  | AT5G18670 | putative beta-amylase BMY3 (BMY3) |
| BN26271 |  |  | 2.14 |  | AT3G55260 | Encodes a protein with β-hexosaminidase activity |
| BN12357, 59 | 2.27 |  | 3.08 |  | AT1G02790 | encodes a exopolygalacturonase |
| BN19117, 18 | 2.86 |  | 2.03 |  | AT4G30280 | XYLOGLUCAN ENDOTRANSGLUCOSYLASE/HYDROLASE 18 (XTH18) |
| BN12646 |  |  | 2.52 | 2.28 | AT1G32170 | xyloglucan endotransglycosylase-related protein (XTR4) |
| BN13628 |  |  | 2.72 |  | AT2G28470 | putative beta-galactosidase (BGAL8 gene) |
| BN18300, 01 |  |  | 2.39 | 2.01 | AT1G35580 | cytosolic invertase 1 (CINV1) |
| BN21422 | 2.44 |  | 3.25 |  | AT3G02570 | maternal effect embryo arrest 31 (MEE31) |
| BN13693 |  |  |  | 3.91 | AT5G18200 | encodes an adenylyltransferase |
| BN22236 |  |  |  | 2.12 | AT4G37800 | xyloglucan:xyloglucosyl transferase, putative / xyloglucan endotransglycosylase, putative / endo-xyloglucan transferase, putative |
| BN26216 |  |  |  | 2.06 | AT4G19810 | glycosyl hydrolase family 18 protein |
| BN22609 |  |  |  | 2.53 | AT4G23820 | glycoside hydrolase family 28 protein / polygalacturonase (pectinase) family protein |
| BN20020 |  |  |  | 2.40 | AT3G26720 | glycosyl hydrolase family 38 protein |
| BN25088 | 2.31 |  |  | 2.01 | AT4G00490 | BETA-AMYLASE 2 (BAM2) |
| BN21398 |  |  |  | 2.66 | AT5G51820 | PHOSPHOGLUCOMUTASE (PGM) |
| BN22807 | 3.77 |  |  | 2.20 | AT4G30440 | UDP-D-GLUCURONATE 4-EPIMERASE 1 (GAE1) |
| BN15860 | 2.11 |  |  |  | AT1G43670 | fructose-1,6-bisphosphatase, putative / D-fructose-1,6-bisphosphate 1-phosphohydrolase, putative |
| BN17464 | 2.27 |  |  |  | AT3G61610 | aldose 1-epimerase family protein |
| BN27052 | 3.71 |  |  |  | AT5G11720 | alpha-glucosidase 1 (AGLU1) |
| BN21021 | 2.46 |  |  |  | AT3G17940 | aldose 1-epimerase family protein |
| BN25419 | 2.18 |  |  |  | AT5G49215 | glycoside hydrolase family 28 protein / polygalacturonase (pectinase) family protein |
| BN10671 | 2.51 |  |  |  | AT3G23770 | glycosyl hydrolase family 17 protein |
| BN24099 | 2.01 |  |  |  | AT3G13560 | glycosyl hydrolase family 17 protein |
| BN15349 | 3.87 |  |  |  | AT5G58330 | malate dehydrogenase (NADP), chloroplast, putative |
| BN11216 | 2.32 |  |  |  | AT5G57655 | xylose isomerase family protein |
| BN22103 | 2.40 |  |  |  | AT5G26570 | chloroplastidic phosphoglucan, water dikinase (PWD) which is required for normal degradation of leaf starch in Arabidopsis |
| BN13810 | 5.53 |  |  |  | AT2G44450 | BETA GLUCOSIDASE 15 (BGLU15) |
| BN16608 | 2.30 |  |  |  | AT3G03640 | Encodes beta-glucosidase (GLUC |
| BN21877 | 8.26 |  |  |  | AT2G32860 | BETA GLUCOSIDASE 33 (BGLU33) |
| BN25288 | 2.99 |  |  |  | AT1G55120 | BETA-FRUCTOFURANOSIDASE 5 (ATFRUCT5) |
| BN26271 | 2.0 |  |  |  | AT3G55260 | BETA-HEXOSAMINIDASE 1 (HEXO1) |
| BN23554 | 3.83 |  |  |  | AT4G09020 | ISOAMYLASE 3 (ISA3) |
| BN21398 | 2.97 |  |  |  | AT5G51820 | PHOSPHOGLUCOMUTASE (PGM) |
| BN22807 | 3.77 |  |  |  | AT4G30440 | UDP-D-GLUCURONATE 4-EPIMERASE 1 (GAE1) |
| BN19102 | 3.15 |  |  |  | AT3G23820 | UDP-D-GLUCURONATE 4-EPIMERASE 6 (GAE6) |
| BN24372 | 2.14 |  |  |  | AT4G30290 | XYLOGLUCAN ENDOTRANSGLUCOSYLASE/HYDROLASE 19 (XTH19) |
| BN10778 | 13.39 |  |  |  | AT4G03210 | XYLOGLUCAN ENDOTRANSGLUCOSYLASE/HYDROLASE 9 (XTH9) |
| BN18300 | 2.25 |  |  |  | AT1G35580 | cytosolic invertase 1 (CINV1) |
| BN11902 | 4.30 |  |  |  | AT5G24400 | embryo defective 2024 (emb2024) |
| BN14584 | 2.82 |  |  |  | AT3G55800 | sedoheptulose-bisphosphatase (SBPASE) |
| Total | 52 | 33 | 35 | 29 | 85 |  |
