# Supplementary material for: Transcriptome profiling of Brassica napus stem sections in relation to differences in lignin content
Source: BMC Genomics. 2018 Apr 16;19:255. doi: 10.1186/s12864-018-4645-6 (PMC5903004; doi:10.1186/s12864-018-4645-6)
Supplement: Supplementary file 16 — Table S10. Targeted transcription factors selected for lignin validation using Arabidopsis mutants. (DOCX 25 kb) [file 12864_2018_4645_MOESM16_ESM.docx]

Table 7: Targeted stem transcription factors selected for validation using Arabidopsis mutant analysis

| # | Gene | *B. napus* ID | Arabidopsis homolog | Arabidopsis knockdown Mutants | DH4-YN4 | DH1-YN1 | DH4-DH1 | YN4-YN1 | Lignin  % of Col-0 |
| --- | --- | --- | --- | --- | --- | --- | --- | --- | --- |
| 1 | Zinc finger (C2H2) (BLUEJAY)  Asymmetric cell division | BN21811 | At1g14580 | CS70742 | 2.78/3.63 | 3.79 | 2.44 | 2.06/2.74/  2.69 | ND |
| 2 | Zinc finger (C2H2)  [multicellular organism development](https://www.arabidopsis.org/servlets/TairObject?type=keyword&id=5590),  [trichome differentiation](https://www.arabidopsis.org/servlets/TairObject?type=keyword&id=14232) | BN23147 | At2g41940 | Salk 045674 | 0.08 | 0.15/0.13 | 0.50 | NDE | ND |
| 3 | Zn finger transcription (jumonji family protein) dioxigenase activity | BN18670 | At1g08620.1 | SALK_029530C | NDE | 6.64 | NDE | NDE | 137.38 |
| 4 | Zinc finger (C3H5)  RING/FYVE/PHD superfamily | BN22477 | At5g05830.1 | Salk 090940 | 5.0 | 2.9 | 6.62 | NDE | ND |
| 5 | Zinc finger (C3H5)  RING/U-box superfamily | BN20501 | At2g23780.1 | CS322616 | 3.56 | NDE | 2.23 | 0.41 | ND |
| 6 | Zinc finger (C3H5)  ABA INSENSITIVE RING PROTEIN 3, encodes a ubiquitin E3 ligase LOG2 | BN17974 | At3g09770.2 | CS24715 | 0.1 | NDE | 0.16 | 2.05 | ND |
| 7 | Beta helix loop helix, Brassinosteroid and GA signalling pathway; HBI1, HOMOLOG OF BEE2 INTERACTING WITH IBH 1 | BN15636 | At2g18300 | Salk 090958 | 4.72/6.56 | 0.49 | NDE | 0.25/2.41 | ND |
| 8 | Beta helix loop helix  ABA-RESPONSIVE KINASE SUBSTRATE 2, AKS2; regulation of stomatal movement | BN12702 | At1g05805 | Salk 106364 | 3.23/3.58/  7.30/3.53 | 2.37 | 2.77/2.54 | 2.19 | ND |
| 9 | PIF4 (beta helix loop helix) Defective in red light responses | BN19746 | AT2G43010.2 | SALK_140393C (CS2103234) | 5.22 | 3.74 | NDE | NDE | 80.8 |
| 10 | *Transparent testa 8* (*TT8*) BHLH42 |  | At4g09820 | *CS111* (*tt8-1*) | NDE | NDE | NDE | NDE | 45.25 |
| 11 | Basic-leucine zipper (bZIP) | BN26545 | AT5G44080.1 | CS302967 | 5.69 | 3.74 | 2.05 | NDE | ND |
| 12 | NAC025/NAM | BN13387 | At1g61110 | Salk 060447 | 5.96/11.16 | 7.82/7.20 | 4.64/5.26 | 2.36/2.34 | ND |
| 13 | ANAC081 |  | At5g08790.1 | Salk 015750C | NDE | NDE | NDE | NDE | 113.90 |
| 14 | NAC/NAM  [cellular response to cold](https://www.arabidopsis.org/servlets/TairObject?type=keyword&id=31524) and chitin; [positive regulation of endoplasmic reticulum unfolded protein response](https://www.arabidopsis.org/servlets/TairObject?type=keyword&id=40040) | BN24136 | At3g49530 | CS2102064 | 9.18/9.81 | NDE | NDE | 3.18/3.39 | ND |
| 15 | NAC/NAM molecular link between cold signals and pathogen resistance | BN24136 | AT3G49530.1 | SALK_103823C | 9.8 | NDE | NDE | NDE | 67.54 |
| 16 | NAC/NAM  Transcript up with wounding and abscisic acid. ATAF1 attenuates ABA signaling and synthesis. | BN17366 | At1g01720 | Salk 057618 | NDE | 0.38/0.43 | 0.17 | NDE | ND |
| 17 | AP2/EREB1  Cytokinin response factor 10 | BN16694 | At1g68550 | Salk 052072 | 4.57 | 3.47 | 0.41/0.44 | NDE | ND |
| 18 | AP2/EREB1 (DEAR2, DREB AND EAR MOTIF PROTEIN)  DREB subfamily A-5 of ERF/AP2 transcription factor family; ethylene-activated signaling pathway | BN16341 | At5g67190 | CS491801 | 2.74/8.94 | NDE | NDE | NDE | ND |
| 19 | MYB34  [cellular response to sulfur starvation](http://www.arabidopsis.org/servlets/TairObject?type=keyword&id=27947), [defense response to insect](http://www.arabidopsis.org/servlets/TairObject?type=keyword&id=25064)s, [indole glucosinolate/ tryptophan biosynthesis,](http://www.arabidopsis.org/servlets/TairObject?type=keyword&id=11430) response to JA | BN17384 | At5g60890 | Salk 006901 | 4.33/2.85 | 4.63/3.83 | 2.82/2.14/  2.98/4.27 | NDE | ND |
| 20 | MYB12  Regulates upper flavonoid pathway. Redundant with MYB11 and MYB111. | BN26939 | At2g47460 | CS9815 (*myb12f*) | 6.38 | 3.75/4.12 | NDE | 2.68/2.09/  3.17 | ND |
| 21 | MYB factor |  | At3g24120.2 | Salk 114420C | NDE | NDE | NDE | NDE | 108.20 |
| 22 | WD40 repeat factor |  | At2g26060.1 | Salk 009567 | NDE | NDE | NDE | NDE | 99.67 |
| 23 | Homeobox 5 (HB5) Class 1 HD ZIP, abscisic acid-activated signalling | BN23391 | At5g65310.1 | Salk 078602 | 10.03 | NDE | NDE | NDE | ND |
| 24 | Scarecrow-like transcription factor 5 (SCL5); GRAS family | BN19829 | AT1G50600.1 | SALK_012789C | 7.22/7.22 | NDE | 2.05/2.33 | 2.13/3.59/  3.77 | 75.41 |
| 25 | Glycine-rich RNA-binding 8 (SNP63)  [innate immune response](https://www.arabidopsis.org/servlets/TairObject?type=keyword&id=11128), response to abscisic acid, [[cytokinin](https://www.arabidopsis.org/servlets/TairObject?type=keyword&id=11401), cold](https://www.arabidopsis.org/servlets/TairObject?type=keyword&id=5433), [zinc ion](https://www.arabidopsis.org/servlets/TairObject?type=keyword&id=12087) CCR1, CIRCADIAN RHYTHM | BN11925 | AT4G39260.2 | SALK_047963C | 14.95/20.59  0.11/0.10 | 0.37/0.59 | NDE | 2.19 | 106.0 |

^1^lignin content relative to wild type Col 0. NDE, no differential expression. ND, not determined.
