# Supplementary material for: Transcriptome profiling of Brassica napus stem sections in relation to differences in lignin content
Source: BMC Genomics. 2018 Apr 16;19:255. doi: 10.1186/s12864-018-4645-6 (PMC5903004; doi:10.1186/s12864-018-4645-6)
Supplement: Supplementary file 17 — Table S11. Selected non-TF genes for lignin validation using Arabidopsis mutants. (DOCX 17 kb) [file 12864_2018_4645_MOESM17_ESM.docx]

Table 8: Microarray and lignin data on selected non-TF genes

|  | Gene | *B. napus* oligo ID | Arabidopsis ID | Arabidopsis knockdown mutants | DH4-YN4 | DH1-YN1 | DH4-DH1 | YN4-YN1 | Lignin  % of Col-0^1^ |
| --- | --- | --- | --- | --- | --- | --- | --- | --- | --- |
| 1 | ABC transporter family protein GENERAL CONTROL NON-REPRESSIBLE 5 (GCN5) | BN16898 | AT5G64840.1 | CS311757 | NDE | 0.24  0.37/0.33 | 2.59/2.82 | NDE | ND |
| 2 | Major intrinsic family protein Ar[senite and](http://www.arabidopsis.org/servlets/TairObject?type=keyword&id=5170) [borate transport](http://www.arabidopsis.org/servlets/TairObject?type=keyword&id=13663), [cellular water homeostasis](http://www.arabidopsis.org/servlets/TairObject?type=keyword&id=14015),  [response to arsenic/borate-containing substance](http://www.arabidopsis.org/servlets/TairObject?type=keyword&id=13589). | BN19934 | At4g10380.1 | Salk 122287C [*nip5;1-1*](http://www.arabidopsis.org/servlets/TairObject?type=polyallele&id=500210687) | 3.53/5.08 | NDE | NDE | 2.05/2.06 | 95.41 |
| 3 | Hexose/glucose transporter  putative plastidic protein | BN21112 | AT5G16150.3 | SALK_066365C | 11.46/9.23  0.09/0.07 | 0.43/0.22 | 3.84/3.43  0.49 | NDE | 88.85 |
| 4 | Cell elongation protein / DIMINUTO 1 (DIM1) CABBAGE 1, CBB1, DIM1, DIMINUTIA, DWARF 1, DWF1, ENHANCED VERY-LOW-FLUENCE RESPONSES 1, EVE; mutant has reduced plant height and roots, round, curly, dark-green leaves; slightly delayed flowering; reduced fertility. Conversion of 24-methylenecholesterol to campesterol. [Lignin metabolic process](https://www.arabidopsis.org/servlets/TairObject?type=keyword&id=6182), response to light, [steroid biosynthesis.](https://www.arabidopsis.org/servlets/TairObject?type=keyword&id=7314) | BN14589 | AT3G19820.2 | SALK_006932 | 11.97/8.50/  2.15 | NDE | 3.8/3.97  2.99/3.15 | 2.00/2.27 | 82.62 |
| 5 | Endo-1,4-beta-glucanase (KORRIGAN 1)  Membrane-bound endo-1,4-beta-D-glucanase, involved in cellulose biosynthesis | BN19160, BN19161 | At5g49720.1 | SALK_075812 | 2.66 | 0.31/0.39 | NDE | NDE | 85.25 |
| 6 | Elongation factor 1-beta / EF1BB  Guanine nucleotide exchange factor that plays role in translation elongation. | BN19950 | AT1G30230.1 | SALK_046102C | 4.98/3.93/  3.09/2.26 | NDE | 2.66/3.51 | 2.20/4.26/  2.48/3.29 | 68.85 |
| 7 | GDLS-motif lipase |  | At1g28570.1 | Salk 094513 | NDE | NDE | NDE | NDE | 94.10 |
| 8 | GDLS-motif lipase |  | At1g71250.1 | Salk 034659C | NDE | NDE | NDE | NDE | 112.90 |
| 9 | De-etiolated 3 (Det3) |  | At1g12840.1 | Salk 072056C | NDE | NDE | NDE | NDE | 118.70 |
| 10 | Glycine-rich RBP |  | At4g39260.1 | Salk 047963C | NDE | NDE | NDE | NDE | 106.00 |
| 11 | Chlorophyll A/B |  | At1g29920.1 | Salk 024534 | NDE | NDE | NDE | NDE | 103.20 |
| 12 | GH9B7 |  | At1g75680.1 | Salk 052927 | NDE | NDE | NDE | NDE | 123.70 |

NDE, no differential expression. ND, not determined. ^1^Lignin assay found in Additional file 1.
